# Analysis of early goal-directed enteral nutrition in nagoya university emergency ICU

**DOI:** 10.1186/2197-425X-3-S1-A183

**Published:** 2015-10-01

**Authors:** N Matsuda, T Higashi, H Umino, G Makishi, T Hinoshita, T Yoshida, K Nakahara, Y Shioya, M Nishikimi, Y Aoyama, A Numaguchi

**Affiliations:** Emergency & Critical Care Medicine, Nagoya University Graduate School of Medicine, Nagoya, Japan

## Introduction

We created early goal-directed enteral nutrition protocol in 2010. The purpose of this study is to evaluate the relation between nutrition management methods and the outcome in emergency and medical ICU.

## Methods

We investigated retrospectively the patients hospitalized for 48 hours or more, in the ICU of in Nagoya University hospital in Japan, from May 2011 to March 2014. According to the first nutrition method, the patients were classified into the following three groups: peroral intake group (PO), enteral nutrition group, and parenteral nutrition group (PN). Enteral nutrition group was classified into the following four groups, according to the time to initiate enteral feeding: within 6 hours (EN6), 6 hours or more, and less than 24 hours (EN6-24), 24 hours or more, and less than 48 hours (EN24-48), and 48 hours or more (EN48). We evaluated APACHEII score and mortality in ICU of each group.

## Results

There were 506 cases in the criteria of this study. Enteral nutrition according to EGDN NAGOYA were 260 cases (51.4%) were classified into enteral group: EN6 38 (14.6%), EN6-24 113 (43.5%), EN24-48 79 (30.4%) and EN48 30 (11.5%). The mean of the APACHE II score was 28.6, 29.8, 29.5 and 29.4, and the ICU mortality was 2.6%, 8.8%, 5.1%, 13.3%, respectively. On the other side, in PN, the APACHE II score was 28.1 and its ICU mortality was 15.6%. EN according to EGDN showed the significantly better outcome than PN in spite of the similar ill severity.

## Conclusions

This study showed that beginning enteral nutrition early could lead to good prognosis of critically ill patients.

